# Multiple White Plaques in the Esophagus: A Possible Case of Esophageal Mucosal Alteration Associated With Immune-Related Adverse Events of Immune Checkpoint Inhibitors

**DOI:** 10.7759/cureus.32710

**Published:** 2022-12-19

**Authors:** Masaya Iwamuro, Takehiro Tanaka, Yoshiyasu Kono, Seiji Kawano, Horoyuki Okada

**Affiliations:** 1 Department of Gastroenterology and Hepatology, Okayama University Graduate School of Medicine, Dentistry, and Pharmaceutical Sciences, Okayama, JPN; 2 Department of Pathology, Okayama University Graduate School of Medicine, Dentistry, and Pharmaceutical Sciences, Okayama, JPN

**Keywords:** nivolumab, ipilimumab, immune-related adverse events, immune checkpoint inhibitor, esophagogastroduodenoscopy

## Abstract

We report two cases of multiple white plaques in the esophagus that emerged after the administration of immune checkpoint inhibitors. Both patients developed enterocolitis as immune-related adverse events associated with immune checkpoint inhibitors. Esophagogastroduodenoscopy revealed duodenal involvement and multiple white plaques in the esophagus. A biopsy of the esophagus showed predominant CD3^+^ lymphocyte infiltration, suggesting that esophageal mucosal alterations were associated with immune-related adverse events. In addition, histopathology showed keratinized stratified squamous epithelium in the first case while increased inflammatory cell infiltration in the intraepithelial and subepithelial layers was observed in the second case. These data suggest a different pathogenesis of the multiple esophageal white plaques between the two cases. Although further investigation is needed to elucidate the significance of these observations, recognition of the esophageal plaques may be important for prompt diagnosis of immune-related adverse events when associated with immune checkpoint inhibitors.

## Introduction

Immune checkpoint inhibitors (ICIs) are a widely used novel class of antitumor drugs. Immune cells possess membrane receptors that transmit inhibitory or stimulatory signals from immune checkpoint molecules like programmed death receptor 1 (PD-1), programmed death-ligand 1 (PD-L1), and cytotoxic T-lymphocyte-associated protein 4 (CTLA-4) [1]. ICIs bind to these molecules or their ligands and inhibit immunosuppressive signaling, leading to T-cell activation in tumor cells. Although ICIs are generally less toxic than conventional antitumor drugs [2], checkpoint inhibition may result in a unique spectrum of side effects termed immune-related adverse events (irAEs).

Inflammation often occurs in the colorectum and small intestine after ICI administration [3]. ICI-associated gastrointestinal symptoms include enterocolitis that manifests as diarrhea, increased bowel movement, abdominal pain, mucousy stool, and/or bloody stool. In contrast, the involvement of the upper gastrointestinal tract, particularly the esophagus, has rarely been reported. Herein, we report two cases of irAE enterocolitis where multiple white plaques were detected in the esophagus using esophagogastroduodenoscopy. Immunostaining of the biopsy specimen from the esophageal plaques showed infiltration of lymphocytes, predominantly CD3^+^ cells. Based on the pathological results, we hypothesize that white plaques are probably irAEs-related mucosal alterations in the esophagus. Here, we report two cases focusing on the endoscopic and pathological features of esophageal lesions and discuss the possible mechanisms for the formation of multiple white plaques in the esophagus.

## Case presentation

Case 1

**Figure 1:**
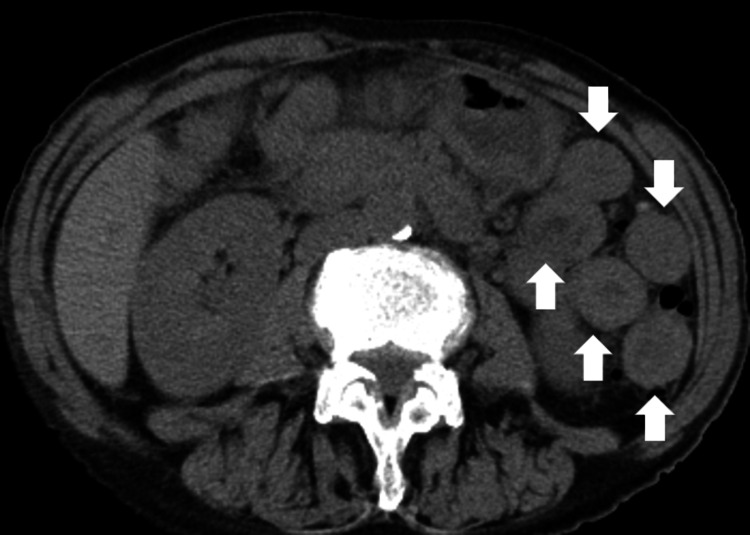
Enterocolitis after avelumab administration in the patient in Case 1 Computed tomography shows diffuse thickening of the small and large intestines (arrows).

A 71-year-old Japanese female patient was diagnosed with muscle-invasive bladder cancer (urothelial carcinoma) with metastasis to the liver, pelvis, and supraclavicular lymph nodes. The patient had a medical history of surgery (vestibular schwannoma at age 44 years) and treatment for eosinophilic chronic rhinosinusitis. She was treated with four cycles of chemotherapy with gemcitabine, cisplatin, and paclitaxel, which resulted in a size reduction in the metastatic tumor. Subsequently, avelumab, a human anti-PD-L1 monoclonal antibody, was administered as a maintenance treatment for metastatic bladder cancer. The patient experienced itching after the second administration of avelumab (45 days after initial administration), followed by diarrhea (4-6 stools per day) after 4 days. Computed tomography (CT) performed 58 days after the initial administration of avelumab revealed diffuse thickening of the small and large intestines (Figure 1, arrows). Her blood test results showed normal white blood cell levels but increased neutrophils, C-reactive protein, lactate dehydrogenase, and creatinine. The levels of hemoglobin, sodium, potassium, and calcium were decreased (Table 1). Liver enzyme levels were within normal ranges. She tested negative for cytomegalovirus antigenemia. The stool was negative for *Clostridium difficile* toxins and antigens. No pathogenic bacteria were detected in stool cultures.

**Table 1:** Significant laboratory results on presentation (Case 1)

Blood test results (units)	Patient value	Reference range
White blood cells (/μL)	7050	3,300–8,600
Neutrophil (%)	70.8	40–70
Hemoglobin (g/dL)	10.2	11.6–14.8
Platelets (/μL)	37.4×10^4^	15.8×10^4^–34.8×10^4^
Total protein (g/dL)	6	6.6–8.1
Albumin (g/dL)	3.3	4.1–5.1
Creatinine (mg/dL)	1.06	0.46–0.79
Lactate dehydrogenase (U/L)	241	124–222
Sodium (mmol/L)	137	138–145
Potassium (mmol/L)	3.1	3.6–4.8
Calcium (mmol/L)	8.6	8.8–10.1
Aspartate aminotransferase (U/L)	15	13–30
Alanine aminotransferase (U/L)	10	7–23
γ-glutamyl transpeptidase (U/L)	24	9–32
Total bilirubin (mg/dL)	0.57	0.40–1.50

**Figure 2:**
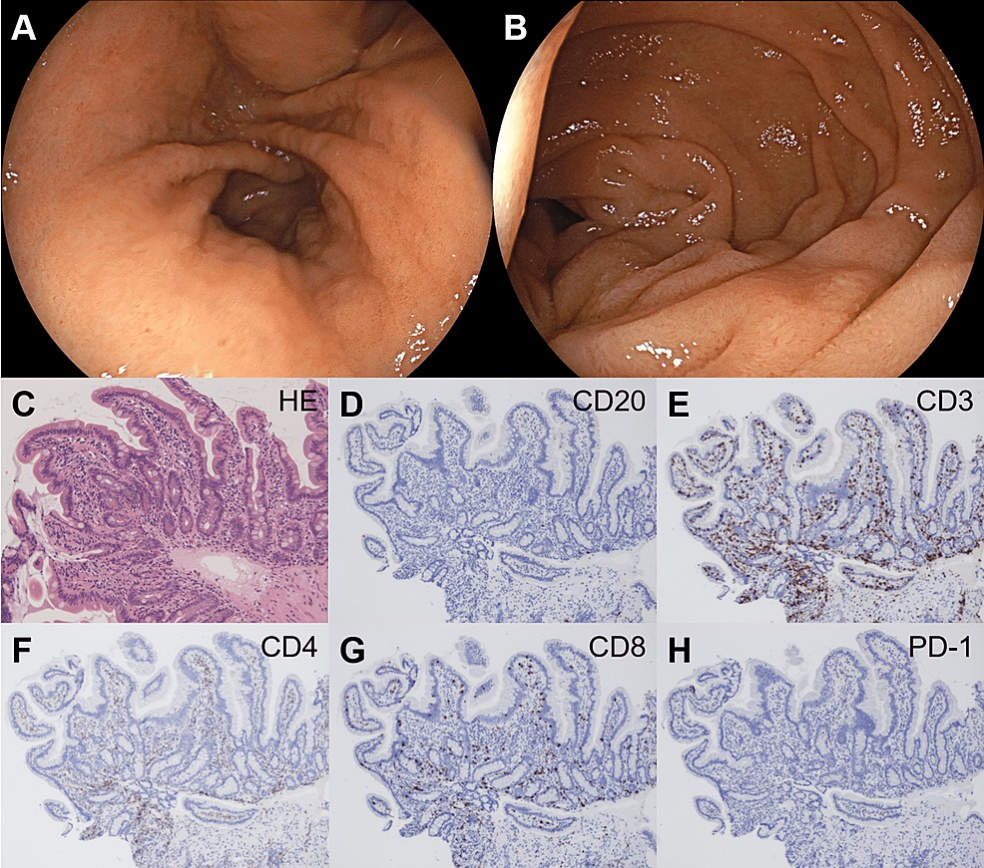
Gastric and duodenal lesions in the patient in Case 1 Esophagogastroduodenoscopy reveals slight stomach erythema (A) and edematous, swelled duodenal mucosa (B). Duodenal biopsy shows the infiltration of mononuclear cells (C). Immunostaining displays that infiltrating cells were negative for CD20 (D) and positive for CD3 (E). CD8^+^ cells (G) outnumbered CD4^+^ cells (F). The cells were negative for PD-1 (H). HE: hematoxylin and eosin stain

Colonoscopy and pathological analysis of the endoscopic biopsy specimens taken from the colorectum indicated no remarkable changes. Esophagogastroduodenoscopy performed the day after admission revealed atrophic gastritis, slight erythema in the gastric antrum (Figure 2A), and edematous and swollen duodenal mucosa (Figure 2B). Biopsy of the duodenum displayed infiltration of mononuclear cells (Figure 2C). Pathologically, the most infiltrated cells were CD3^+^, indicating T lymphocyte predominance (Figure 2E). Limited CD20^+^ cells (Figure 2D) and considerably more CD8^+^ cells than CD4^+^ cells were detected (Figures 2F, 2G). The cells were negative for PD-1 (Figure 2H).

**Figure 3:**
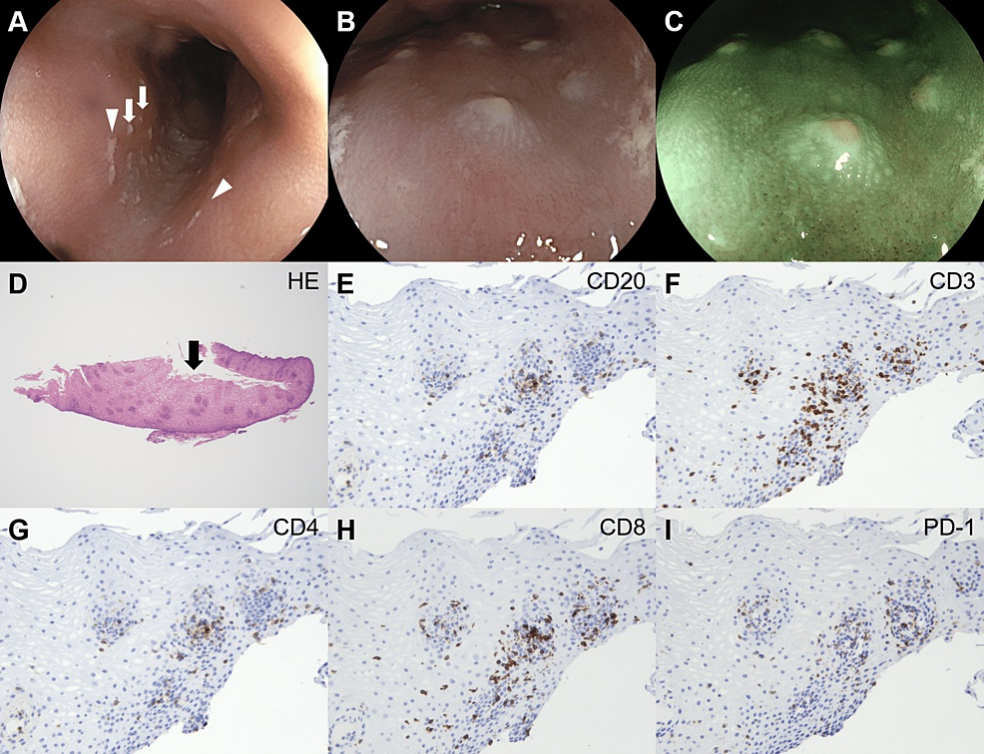
Esophageal lesions in the patient in Case 1. White adhesions (arrowheads) and plaques (arrows) are observed in the esophagus (A). Magnifying observation reveals round, white esophageal plaques (B, white light; C, blue laser imaging). Esophageal biopsy shows keratinized stratified squamous epithelium (D, arrow). Mononuclear cell infiltration is observed in the epithelial layer. Most of the infiltrated cells are positive for CD3 (F) and CD8 (H) and partly positive for CD20 (E), CD4 (G), and PD-1 (I). HE: hematoxylin and eosin stain.

Esophagogastroduodenoscopy revealed white adhesions and plaques in the esophagus (Figure 3A). Although the white adhesions (Figure 3A, arrowheads) were removed after lavage with a water jet flow via the endoscope, the white plaques (Figure 3A, arrows) did not detach. Magnified observation displayed the morphology of round white plaques (Figure 3B, white light; Figure 3C, blue laser imaging). A biopsy of the esophageal lesion sample revealed keratinized stratified squamous epithelium (Figure 3D, arrow). Although mononuclear cells infiltrated the epithelial layer, they were absent from the keratinized area. Most infiltrated cells were positive for CD3 (Figure 3F) and CD8 (Figure 3H) and partly positive for CD20 (Figure 3E), CD4 (Figure 3G), and PD-1 (Figure 3I).

**Figure 4:**
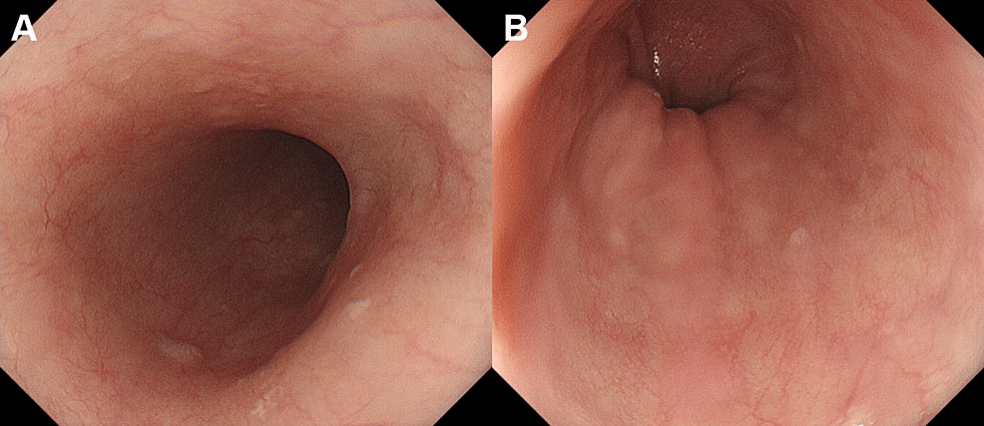
Esophagogastroduodenoscopy images of the patient in Case 1 after corticosteroid administration White plaques in the esophagus have disappeared.

Even though apoptosis was not detected in the intestinal epithelium in the biopsy specimens, the patient was diagnosed with ICI-induced enteritis based on her symptoms and CT findings. She was treated with an intravenous corticosteroid (50 mg/day of prednisolone) that resolved her diarrhea, as well as the duodenal wall thickening. Esophagogastroduodenoscopy performed eight days after corticosteroid administration showed the disappearance of white plaques from the esophagus (Figure 4). Avelumab treatment was terminated, and chemotherapy with gemcitabine, cisplatin, and paclitaxel was initiated.

Case 2

A 54-year-old Japanese female patient noticed black pigmentation on her right heel and assuming an ulcer, the patient visited a dermatologist. Malignant melanoma was diagnosed after surgical resection of the heel ulcer. Despite a negative surgical margin on pathological analysis, sentinel lymph node biopsy revealed metastasis to popliteal lymph nodes. Nivolumab, a human anti-PD-1 monoclonal antibody, was administered for one year as adjuvant therapy for metastatic malignant melanoma. However, enlargement of the right inguinal and external iliac lymph nodes was noticed after 17 months of diagnosis, and malignant melanoma metastasis was diagnosed after lymph node dissection. Even after pembrolizumab was administered three times, multiple lung and liver metastases were observed. Therefore, combination immunotherapy with nivolumab and ipilimumab, a human anti-CTLA-4 monoclonal antibody, was initiated. The patient was admitted to another hospital 24 days after the administration of combination immunotherapy because of fever, watery stool (4-6 stools per day), cough, and general malaise. A colonoscopy revealed no colorectal abnormalities. The patient tested negative for cytomegalovirus antigenemia and *Clostridium difficile* toxin. Pathogenic bacteria were absent in the stool culture. As her symptoms showed no improvement, with the appearance of vomiting, the patient was transferred to our hospital 40 days after the administration of combination immunotherapy.

**Figure 5:**
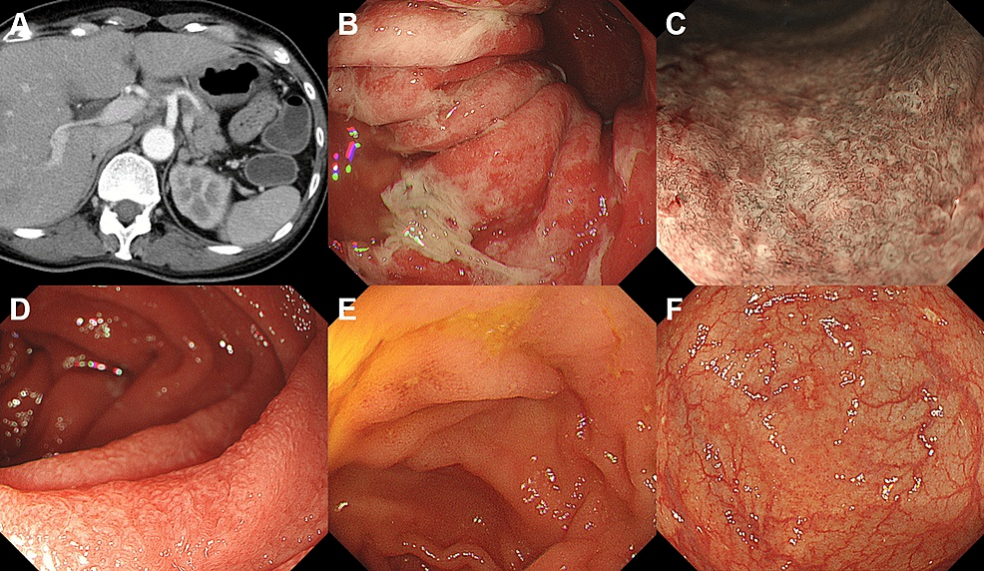
Immune checkpoint inhibitors-induced gastrointestinal immune-related adverse events in the patient in Case 2 After combination immunotherapy with nivolumab and ipilimumab, computed tomography shows fluid retention in the colon (A). Esophagogastroduodenoscopy reveals fibrin exudate in the stomach (B). Magnifying observation with narrow-band imaging shows the destruction of the glandular structure of the gastric mucosa (C). Duodenal mucosa is atrophic (D). No consequent esophageal abnormalities are observed. Colonoscopy shows small erosions in the ileum (E) and colon (F).

CT revealed fluid retention in the colon (Figure 5A). Esophagogastroduodenoscopy performed the day after admission revealed fibrin exudate in the gastric mucosa (Figure 5B). Magnifying observations with narrow-band imaging displayed considerable destruction of the glandular structure of the gastric mucosa (Figure 5C) and an atrophic duodenal mucosa (Figure 5D) but no esophageal abnormalities. a colonoscopy revealed small erosions in the ileum (Figure 5E), cecum, and colon (Figure 5F). Duodenal biopsy showed active inflammation with villous atrophy, dense mononuclear infiltration, and duct destruction, leading to the diagnosis of irAEs. The patient was treated with intravenous corticosteroids (prednisolone 50 mg/day) and proton pump inhibitors, and consequent relief from diarrhea was seen.

**Figure 6:**
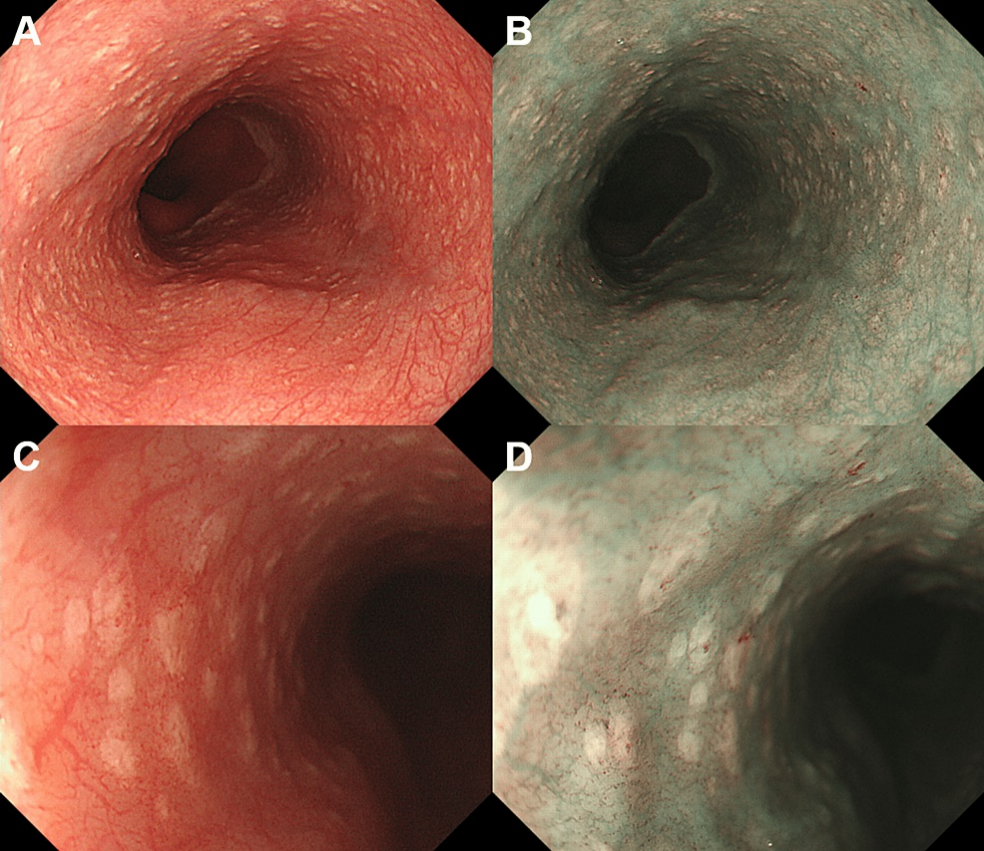
Esophagogastroduodenoscopy of the patient in Case 2 Multiple thin, white plaques are observed in the esophagus. (A) white light observation; (B) narrow-band imaging; (C) magnifying observation with white light; (D) magnifying observation with narrow-band imaging

Esophagogastroduodenoscopy performed seven days after corticosteroid administration revealed partial improvement in inflammation in the stomach and duodenum area, but multiple thin white plaques emerged in the esophagus (Figure 6).

**Figure 7:**
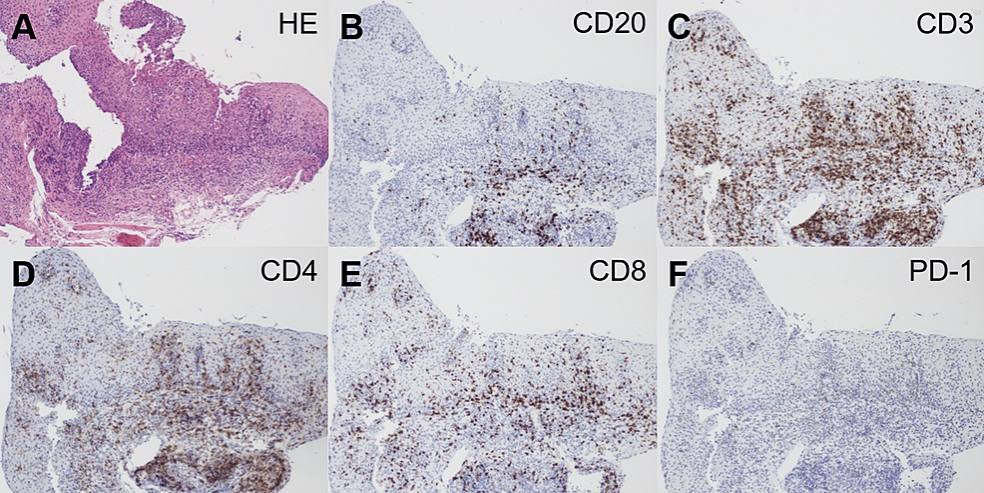
Histopathology of the esophagus in the patient in Case 2 A biopsy of the white plaques shows inflammatory cell infiltration in the intraepithelial and subepithelial layers, squamous epithelium thickening, and edema (A). The infiltrated cells are positive for CD3 (C), partially positive for CD20 (B), and negative for PD-1 (F). CD4^+^ cells (D) outnumber CD8^+^ cells (E). HE: hematoxylin and eosin stain

A biopsy of these white plaques showed inflammatory cell infiltration in the intraepithelial and subepithelial layers, thickening of the squamous epithelium, edema, and increased density of capillary vessels (Figure 7A). Immunostaining revealed that most of the infiltrated cells were positive for CD3 (Figure 7C), partially positive for CD20 (Figure 7B), and negative for PD-1 (Figure 7F). Although both CD4^+^ (Figure 7D) and CD8^+^ cells were present (Figure 7E), CD4^+^ cells were predominant.

## Discussion

In our report, we describe two patient cases where diarrhea occurred after ICIs administration. Based on their symptoms and radiologic features, the patients were diagnosed with irAE enteritis. Diarrhea due to enterocolitis is a well-established ICI-induced adverse event. Wang et al. retrospectively investigated 327 cancer patients who received ICIs and reported that 117 patients (35.7%) had diarrhea and 79 (24.2%) required immunosuppressive treatment with systemic corticosteroids alone or corticosteroids in combination with infliximab [4]. The use of the anti-CTLA-4 monoclonal antibody reportedly increased the risk of enterocolitis compared with when anti-PD-1 or PD-L-1 monoclonal antibodies were administered, although the latter is also moderately associated with gastrointestinal toxicities [5-7]. Since the present patients had grade 2 diarrhea (4-6 stools per day above baseline), ICI therapy was withheld and corticosteroids were initiated after infectious causes were ruled out [8]. In addition to enteritis or enterocolitis, the stomach (Case 2) and duodenum (Cases 1 and 2) were also involved in these cases. Because of this, we consider esophagogastroduodenoscopy to be valuable for patients with gastrointestinal symptoms after ICI use, particularly when colonoscopy shows only subtle or no abnormalities.

As seen in the esophagogastroduodenoscopy images, multiple white plaques emerged in the esophagus during the course of irAE enteritis or enterocolitis in both patients; these plaques disappeared in the irAE patient after steroid administration (Case 1). Because of this, the presence of multiple white plaques in the esophagus was considered a possible mucosal alteration associated with irAEs. Interestingly, esophageal involvement with irAEs is infrequently reported, but cases of multiple ulcers as typical features of esophageal lesions exist [9-13]. Other endoscopic features of esophageal manifestations of irAEs include distal esophagitis resembling Barrett’s esophagus [14], thick mucoid secretions, diffuse mucosal congestion with edema, erythema, mucosal friability [15], and ulcers with desquamative esophagitis in the distal esophagus [16]. However, multiple white esophageal plaques have not been previously reported.

In the present cases, immunostaining of the biopsy specimen displayed a predominance of CD3+ cells, with marginal CD20+ cells, indicating that the majority of infiltrating cells were T lymphocytes. In addition, while most cells were CD8+ in the first case, CD4+ cells (>CD8+ cells) were predominant in the second case. An increased number of CD8+ cells is a pathological feature of anti-PD-1 and anti-PD-L1 monoclonal antibody-associated irAEs while CD4+ cells outnumber CD8+ cells in patients with anti-CTLA-4 monoclonal antibody-associated irAEs [17] as in our case.

Although both patients presented with multiple white esophageal plaques, we speculate that the pathogenesis is different. In the second case, a biopsy revealed inflammatory cell infiltration in the intraepithelial and subepithelial layers, squamous epithelium thickening, and mucosal edema. We consider that the increased cellularity of the esophageal mucosa due to infiltration by mononuclear cells was visualized as white plaques. In contrast, the keratinized stratified squamous epithelium was observed in the first case, and minimal inflammatory cell infiltration was observed. Furthermore, esophageal hyperkeratosis also presents as a white plaque-like lesion [18]; therefore, the increased thickness of the keratinized stratified squamous epithelium could have been endoscopically observed as white plaques. These differences in pathological features might have caused the differences in endoscopic images between the two patients; magnifying observation could reveal tiny white depositions in the first patient (Figures 3B, 3C) and multiple small white membranous lesions in the second patient (Figures 6C, 6D).

Corticosteroids are the most commonly used treatment for irAEs. Infliximab and vedolizumab are also used for steroid-refractory colitis associated with ICIs [19]. Steroids have also been used for ICI-related esophageal lesions in previous reports [9-12]. Proton pump inhibitors or vonoprazan have been prescribed independently [13] or in combination with steroids [9,12] for the treatment of esophageal ulcers because acid reflux theoretically causes further deterioration of mucosal damage in the esophagus. Appropriate management of esophageal white plaques such as those seen in the present cases, however, has not yet been determined, likely because these lesions do not appear to further deteriorate the patient’s health.

Finally, this is the first report that describes white plaques as possible esophageal mucosal alterations associated with ICIs. However, their clinical significance remains uncertain. To date, major gastrointestinal toxicities reported with ICI use include colitis, hepatitis, gastritis, and enterocolitis, whereas esophageal involvement has not attracted much attention [20]. The grading of ICI-induced colitis is well-established; an increase of < 4 stools per day over baseline is defined as Grade 1, an increase of 4-6 stools per day over baseline is defined as Grade 2, an increase of ≥ 7 stools per day over baseline, incontinence, hospitalization, and limiting self-care activities during daily living are defined as Grade 3, and life-threatening consequences and urgent intervention are defined as Grade 4 [20]. In contrast, the classification of severity in the upper gastrointestinal tract, (the esophagus, stomach, and duodenum) has not yet been established. An increasing number of cases where patients developed esophageal lesions during ICI use would facilitate understanding of the significance of this condition and allow grading of this disease. We believe that identification and biopsy sampling of these lesions may aid physicians in promptly diagnosing irAEs; thus, careful observation of the esophagus, stomach, and duodenum during esophagogastroduodenoscopy in patients receiving ICIs is important. As multiple white plaque-like lesions and exudates adherent to the esophageal mucosa are often identified as manifestations of infectious esophageal candidiasis [21], and whitish protrusions should be differentiated from esophageal papilloma [22], endoscopists should be informed that white plaques may be alternatively associated with ICI use.

## Conclusions

We present two case studies of patients with white esophageal plaques that were treated with ICIs. These cases highlight the importance of being aware of and recognizing multiple white plaques in the esophagus in patients who are treated with immune checkpoint inhibitors. Although further investigation is required, recognition of these endoscopic features may be important for the prompt diagnosis of irAEs.
